# 25-Hydroxyvitamin D and Peripheral Immune Mediators: Results from Two Nationwide Danish Pediatric Cohorts

**DOI:** 10.3390/nu9040365

**Published:** 2017-04-06

**Authors:** Steffen U. Thorsen, Christian B. Pipper, Kristin Skogstrand, Flemming Pociot, Jannet Svensson

**Affiliations:** 1 Copenhagen Diabetes Research Center (CPH-DIRECT), Department of Pediatrics, Herlev Hospital, University of Copenhagen, Herlev Ringvej 75, 2730 Herlev, Denmark; flemming.pociot.01@regionh.dk (F.P.); Jannet.Svensson@regionh.dk (J.S.); 2 Department of Public Health, Section of Biostatistics, University of Copenhagen, Copenhagen, Oester Farimagsgade 5, 1710 Copenhagen K, Denmark; pipper@sund.ku.dk; 3 Department of Congenital Disorders, Center for Neonatal Screening, Statens Serum Institute, Artillerivej 5, 2300 Copenhagen S, Denmark; ksk@ssi.dk; 4 Department of Clinical Medicine, Faculty of Health and Medical Sciences, University of Copenhagen, Blegdamsvej 3B, 2200 Copenhagen N, Denmark

**Keywords:** Diabetes Mellitus, Type 1, vitamin D, Cytokines/immunology, TREM1, human, C-reactive protein, mannose-binding lectin, Infant, Newborn, Child, Adolescent

## Abstract

(1) Background: We aimed to examine if 25-hydroxyvitamin D (25(OH)D) was related to the peripheral immunological and inflammatory signature both at birth, and in newly diagnosed patients with childhood type 1 diabetes (T1D) and their healthy controls; (2) Methods: The birth cohort consisted of 470 patients and 500 healthy controls. Dried blood samples were collected from the neonates in the period 1981–1999. The newly diagnosed cohort consisted of 460 patients and 453 siblings. Serum samples were collected in the period 1997–2005. A variety of peripheral immune mediators were measured and compared to total 25(OH)D levels (25(OH)D_2_ + 25(OH)D_3_). For each immune mediator, the relative change (RC) in the mean level was modeled by robust log-normal regression and correction for multiple testing was performed; (3) Results: Two associations were identified; there was a negative association between 25(OH)D (10 nmol/L increase) and leptin (RC (95% confidence interval (CI)), 0.98 (0.96; 1.00)), and a positive association between 25(OH)D (10 nmol/L increase) and the chemokine, chemokine (c-x-c motif) ligand (CXCL) 8 (RC (95% CI), 1.07 (1.01; 1.13)); (4) Conclusion: CXCL8 and leptin have significant associations with levels of 25(OH)D in the newly diagnosed cohort. These results do not indicate a strong influence of 25(OH)D on the peripheral immunological or inflammatory signature.

## 1. Introduction

Type 1 diabetes (T1D) is an endocrine disease that results from autoimmune destruction of the pancreatic insulin-producing β-cells, leading to a loss of insulin secretion and symptomatic hyperglycemia. Changes in the complex interplay between genetic, epigenetic, and environmental factors are thought to be involved in T1D etiopathogenesis [1,2,3]. 

Vitamin D is in the spotlight, as many in vitro and animal studies indicate that vitamin D has anti-inflammatory activity and may also prevent or halt the destruction of β-cells [4,5]. Findings from epidemiological studies focusing on the association between vitamin D and T1D, both in early life and around time of diagnosis, are inconsistent [6,7]. However, to our knowledge, no large-scale studies have yet examined if there are any associations of 25-hydroxyvitamin D (25(OH)D) with peripheral immune mediators in cohorts of patients with childhood T1D and healthy controls. Therefore, we aimed to examine if 25(OH)D was associated with a wide range of peripheral immune mediators at two different time-windows, namely (1) at birth in patients who subsequently developed childhood T1D and their healthy controls and (2) in newly diagnosed patients with childhood T1D, and their healthy siblings. 

## 2. Materials and Methods

### 2.1. Study Design, Sample Population, Data Sources and Variables

We conducted two separate case-control studies—both study samples are described extensively elsewhere including coding of covariates [7,8,9,10]. 

#### 2.1.1. The Birth Cohort Samples

Blood sampling (dried blood spots (DBS)) was performed within a week following birth during the years 1981 to 1999. For each identified clinical case (a neonate who subsequently develops T1D before the age of 18 years) one control was selected based on having measurements on both peripheral immune mediators and 25(OH)D. Clinical cases were identified from the Danish Patient Register and further validated against the Danish Registry of Childhood and Adolescent Diabetes (DanDiabKids) [11]. Our random sample of clinical cases and controls are based on a sub-sample from a population-based case-control study comprised of 2086 clinical cases and 4172 controls [12]. In the original study the following inclusion criteria were: (1) born between 1981 and 2002 (<0.5% of the individuals were born between 2000 and 2002); (2) diagnosed with T1D between 1 January 1981 and 1 May 2004; (3) had an available DBS card in the Danish Newborn Screening Biobank (DNSB). Exclusion criteria were: (1) additionally diagnosed with another diabetes type; (2) insufficient biological material on the DBS card; (3) not a singleton (3% of the cohort). Controls were selected by collecting the DBS card next to the case’s card in the DNSB, hereby matching on date of birth.

Since 1981 the DBS have been stored at −20 °C/−4 °F in the DNSB and this biobank comprises almost 100% of the Danish population born since 1982 [13].

#### 2.1.2. The Newly Diagnosed Cohort Samples

A random sample of 500 clinical cases was collected from a biobank linked to the DanDiabKids. Blood samples were taken less than three months after the onset of T1D during the years 1997 to 2005. The onset date was defined as the date of the first insulin injection. The control group comprised a random sample of 500 siblings selected from an eligible sample of approximately 2000 siblings with blood samples stored in the biobank and a sibling diagnosed with T1D. The chosen control sample was independent of participation of a sibling with T1D in the study. Randomization aimed at covering all sample months (January 1997 to December 2005). A last inclusion criterion was that all individuals had to be between 0 to 18 years of age at time of blood sampling. 

Serum samples from the DanDiabKids were stored at −80 °C/−112 °F during the entire study period from 1997 to 2005.

### 2.2. Outcome Assessment 

#### 2.2.1. Assessment of Peripheral Immune Mediators on Dried Blood Spots

Multiplexed sandwich immunoassays and the flowmetric Luminex xMAP^®^ technology were used to quantify the following cytokines: interleukin (IL)-1β, IL-4, IL-6, chemokine (c-x-c motif) ligand 8 (CXCL8), IL-10, IL-12 (p70), interferon gamma (IFNγ), tumor necrosis factor alpha (TNFα), transforming growth factor beta 1 (active form) (TGFβ), leptin, and adiponectin. In addition, we also quantified c-reactive protein (CRP), mannose-binding lectin (MBL) and soluble triggering receptor expressed on myeloid cells-1 (sTREM-1) [14]. In all assays, matched pairs were run together to avoid batch effects/interassay variation [15]. Biomarker analyses are described in detail elsewhere [14]. 

Quality control of the analysis were made using mouse IL-6 as an internal analyte added to the extraction buffer to detect pipetting errors, and biotinylated beads to detect signal errors (more thoroughly described in Skogstrand et al. [16]). Calibration curves were used on each plate together with one high and two low controls. Samples, calibrators, and controls were analyzed in duplicate.

#### 2.2.2. Assessment of Peripheral Immune Mediators in Serum

The serum samples were analyzed using the commercially available high-capacity Luminex xMAP technology combined with a 15-plex and 3-plex developed in-house for the simultaneous determination of 18 biomarkers, as described in Skogstrand et al. [14]. The secreted immune and inflammatory mediators measured were: IL-1β, IL-4, CXCL8, IL-10, IL-12 (p70), IL-18, IFNγ, TNFα, TGFβ, CCL2, CCL3, CCL4, CCL5, leptin, adiponectin, CRP, MBL and sTREM-1. The coefficient of variation (CV), as described elsewhere [14], was analysed for each of the assays. 

### 2.3. Exposure Assessment 

#### 2.3.1. Assessment of 25(OH)D on Dried Blood Spots

In short, 25(OH)D status was assessed by measuring 25(OH)D_2_ and 25(OH)D_3_ in 3.2 mm samples (also called punches) taken from DBS cards. Sample preparation and analysis was performed using liquid chromatography–mass spectrometry (LC-MS) according to a modified method [17], described in detail elsewhere [9]. 

The lower limit of quantification (LLOQ) was 4 nmol/L for 25(OH)D_3_ and 3 nmol/L for 25(OH)D_2_. The 25(OH)D levels from the DBS are full blood concentrations. Most 25(OH)D molecules in the bloodstream are protein bound and to approximate and report sera concentrations we corrected the original levels using the formula: serum 25(OH)D = full blood 25(OH)D × (1/(1 − 0.61)), where 0.61 is the haematocrit fraction for capillary blood [18].

#### 2.3.2. Assessment of 25(OH)D in Serum

Vitamin D status was measured as serum 25(OH)D by high-performance liquid chromatography (HPLC) [19]. Detection limit for 25(OH)D was 9.5 nmol/L, with a CV of 8%.

### 2.4. Statistical Analysis

For each peripheral immune mediator the relative change (RC) in the mean level by 10 nmol/L increase in total 25(OH)D (equivalent to 25(OH)D_2_ + 25(OH)D_3_) was modeled by a robust log-normal model regression, which takes into account: (1) that measurements are potentially both left and right censored; and (2) correlation within assay. To account for correlation within cluster inference was based on a working independence generalized estimation equation (GEE) approach. For the birth cohort the clusters are equivalent to assay. For the newly diagnosed cohort, the cluster was equivalent to family/sibling ID.

For the birth cohort, the following risk factors are included in the model: 25(OH) levels, sex, case status (T1D or control), gestational age, mothers age, birth weight, season and calendar year group. For the newly diagnosed cohort the following risk factors are included in the model: 25(OH) level, sex, case status (T1D or sibling), age at blood sampling, season and calendar year group. The coding of these variables is presented in Table 1. 

Simultaneous evaluation of risk factors on all peripheral immune mediators was done using the model stacking approach detailed in Pipper et al. [20]. Subsequent adjustment for multiple testing and familywise 95% confidence bands are calculated using the single step procedure by Hothorn et al. [21]. Likelihood ratio estimates of mean ratios and accompanying confidence limits are calculated on a log scale and transformed back to the original scale. 

Overall functional misspecification by including 25(OH)D as a trend (linear variable) was assessed by a lack-of-fit test. Specifically, we included a quadratic term of 25(OH)D and tested its significance by a robust Wald test. 

All analyses are made using the statistical software package R version 3.2.0 (the R foundation for statistical programming, Vienna, Austria) and the add-on packages survival, ggplot2, and multcomp.

### 2.5. Ethics

Both studies were performed in accordance with the Helsinki II Declaration. Furthermore, both studies were approved by the Danish Ethical Committee (H-4-2013-049 and H-KA-20070009). All of the patients and their parents or guardians gave informed consent.

## 3. Results

### 3.1. Basic Characteristics for both Cohorts are Presented in Table 1

The birth cohort contains 970 individuals with a complete set of covariates (470 patients and 500 controls). In the birth cohort, the median/first and third quartile (Q1–Q3) 25(OH)D level was 26.3/17.1–38.8 nmol/L for patients and 25.7/17.0–37.8 for controls. The newly diagnosed cohort contains 913 individuals with a complete set of covariates (460 patients and 453 controls). In the newly diagnosed cohort the median/Q1–Q3 25(OH)D level was 62.0/40.1–95.5 for patients and 57.9/38.0–89.0 nmol/L for controls. It is noteworthy to mention that we have already shown that 25(OH)D levels are not associated with later risk of childhood T1D in the birth cohort and levels between newly diagnosed patients with childhood T1D and their healthy siblings do not differ either [7,9]. Levels of the peripheral immune mediators are presented in Table 2.

### 3.2. Association between Peripheral Immune Mediators and 25(OH)D in the Birth Cohort

**Figure 1:**
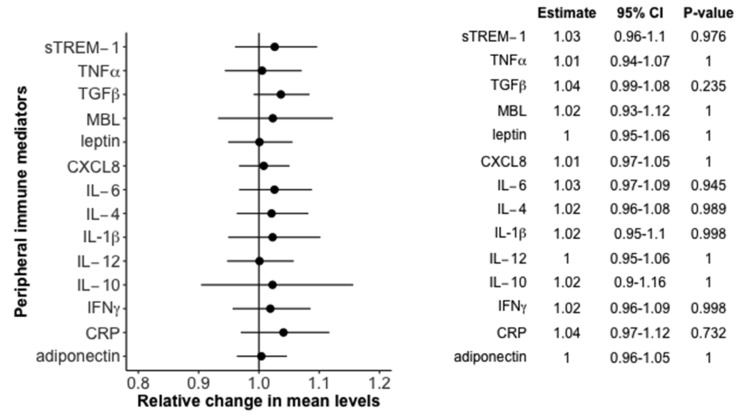
Relative change in mean levels of immune mediators with 95% confidence bands by 10 nmol/L increase in 25(OH)D levels in the birth cohort—results from the full adjusted model. IL, interleukin; IFNγ, interferon gamma; TNFα, tumor necrosis factor alpha; TGFβ, transforming growth factor beta; CRP, c-reactive protein; MBL, mannose-binding lectin; sTREM-1, soluble triggering receptor expressed on myeloid cells-1; CXCL, chemokine (c-x-c motif) ligand.

We were unable to detect any linear association of 25(OH)D on the 14 examined peripheral immune mediators. The numeric results including a graphical overview (forest plot) are presented in Figure 1.

We also examined if non-linear associations existed between 25(OH)D and the peripheral immune mediators in the birth cohort, but found no sign of such associations (*p* = 0.25).

### 3.3. Association between Peripheral Immune Mediators and 25(OH)D in the Newly Diagnosed Cohort

**Figure 2:**
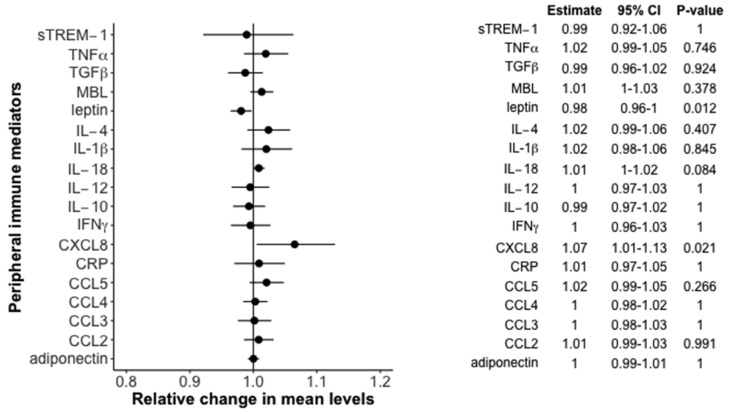
Relative change in mean levels of immune mediators with 95% confidence bands by 10 nmol/L increase in 25(OH)D levels in the newly diagnosed cohort—results from the full adjusted model. IL, interleukin; IFNγ, interferon gamma; TNFα, tumor necrosis factor alpha; TGFβ, transforming growth factor beta; CRP, c-reactive protein; MBL, mannose-binding lectin; sTREM-1, soluble triggering receptor expressed on myeloid cells-1; CXCL, chemokine (c-x-c motif) ligand; CCL, chemokine (c-c motif) ligand; CI, confidence interval.

We only found a statistical significant positive association between 25(OH)D (10 nmol/L increase) and CXCL8 (RC (95% CI), 1.07 (1.01; 1.13), *p*_adjusted_ = 0.02), and a negative association between 25(OH)D (10 nmol/L increase) and leptin (RC (95% CI), 0.98 (0.96; 1.00), *p*_adjusted_ = 0.01). The other 16 peripheral immune mediators were not associated with 25(OH)D. The numeric results including a graphical overview (forest plot) are presented in Figure 2.

As for the birth cohort, we also examined if a non-linear associations between 25(OH)D and the peripheral immune mediators existed in the newly diagnosed cohort, but no proof of such associations were found (*p* = 0.26).

## 4. Discussion

In these two large-scale, case-control studies, 14 peripheral immune mediators were measured in a birth cohort and 18 peripheral immune mediators were measured in a newly diagnosed childhood T1D cohort. 25(OH)D levels were compared with these immune and inflammatory factors, and two peripheral immune mediators, i.e., CXCL8 and leptin, were associated with 25(OH)D in the newly diagnosed cohort. These results do not indicate a strong role of 25(OH)D as an immune modulator when using peripheral immune mediators as a proxy for the child’s immunological fingerprint. 

We demonstrated that CXCL8 levels in our newly diagnosed cohort rises with 1%–13% for every 10 nmol/L increase in 25(OH)D. CXCL8, also known as IL-8, is a chemokine which affects leukocyte migration positively and thereby promotes inflammation [22]. Interestingly, the CXCL8 gene has been shown to be a vitamin D receptor (VDR) binding-site; hence the active form of vitamin D (1,25(OH)_2_D) can alter, e.g., up-regulate, its expression, which may help to clear infections by recruitment of specific immune cells [23,24]. In an in vitro study using hyperinflammatory macrophages from patients with cystic fibrosis, vitamin metabolites have been shown, in high concentrations (e.g., 25(OH)D >100 nmol/L), to down-regulate CXCL8 [25]. These discrepancies could be due to different cell lines used e.g., monocytic leukemia cell line (THP-1) versus hyperinflammatory macrophages, but may also depend on choice of vitamin D metabolite, vitamin D metabolite concentration, and cell culture duration [23,25]. Our results reflect an overall systemic in vivo association of 25(OH)D on CXCL8 levels. The role of CXCL8 in regards to T1D pathogenesis remains to be examined, but we previously reported no difference in CXCL8 levels between patients and healthy controls in both these cohorts [26]. Furthermore, no association between 25(OH)D and CXCL8 was found in the birth cohort.

We also found leptin levels significantly reduced with 0%–4% for every 10 nmol/L increase in 25(OH)D in the newly diagnosed cohort. Leptin is an adipokine, secreted primarily from adipose tissue. The overall action of leptin in the immune system is to activate leukocytes and mediate inflammation [27]. In a recent study that utilized the newly diagnosed cohort, we found leptin levels were 10%–40% lower in patients with childhood T1D compared to their healthy siblings, but this finding was not mirrored by higher 25(OH)D levels [7,28]. A systematic review and meta-analysis conducted on adult populations without T1D also found evidence of an inverse relationship between 25(OH)D and leptin, but well-designed clinical trials with vitamin D supplementation of more than 1000 IU/day are needed to confirm such a relationship [29].

The effect sizes of the abovementioned significant associations are both small, and may not have profound immunological importance. Importantly, we do not find any association between physiological 25(OH)D levels and well-known Th1, Th2 or Treg cytokines that have been associated with either β-cell destruction or protection e.g., IL-1β, IFNγ, TNFα, IL-10 and TGFβ [30,31]. 

This study has a number of strengths. Firstly, the sample size in both cohorts includes over 900 individuals—both cohorts consist of a patient group (childhood T1D) and a healthy control group (in the newly diagnosed cohorts these healthy controls are a sampling of siblings). Secondly, both cohorts are population-based and patients with childhood T1D are thoroughly validated and controls are randomly selected. Thirdly, quantification of both 25(OH)D and a broad spectrum of peripheral immune mediators, initially measured for testing other hypotheses, gave us the opportunity to conduct the current study using these unique cohorts. Our study also has some limitations. Firstly, a single measurement of both 25(OH)D and peripheral immune mediators may not detect long-term differences or reflect dynamic changes. Secondly, sample storage time may influence assay measures due to degradation, however calendar year of blood sampling was included in both models to control for this. Thirdly, one may ask if these measured peripheral immune mediators, in some part, mirror or affect the tissue-specific immunological micro-milieu in the islet of Langerhans in the pancreas. Or, it could be the case that we just looking at a more general immunological cross-sectional fingerprint and its association with 25(OH)D. Either way, we find a very small influence of 25(OH)D on these peripheral immune mediators. 

## 5. Conclusions

We have examined a wide range of peripheral immune and inflammatory mediators in both neonates who subsequently develop childhood T1D, and in a separate cohort of newly diagnosed patients with childhood T1D. Two immune factors, i.e., CXCL8 and leptin, have significant associations with levels of 25(OH)D in the newly diagnosed cohort. These results indicate that vitamin D does not appear to play a major role as an immune-modulator of the peripheral immune system. To further understand a possible role of vitamin D on the human immune system, studies need to be performed in more complex systems that include genetics, repeated measures of functional immune cell populations and gene-immune mediator interactions. 

## Figures and Tables

**Table 1:** Descriptive characteristics of the two pediatric cohorts.

Variables	Birth Cohort			Newly Diagnosed Cohort		
	Patient	Control	*p*-Value ^1^	Patient	Sibling	*p*-Value
	(*n* = 470)	(*n* = 500)		(*n* = 460)	(*n* = 453)	
Basic characteristics						
Sex						
Female, *n*/% of total	231/49.1	231/46.2	0.39	218/47.4	198/43.7	0.29
Male, *n*/% of total	239/50.9	269/53.8		242/52.6	255/56.3	
Age at onset ^2^						
Median/	8.2/			10.4/	10.3/	0.41
Q1–Q3 ^3^, years	4.8–11.0			7.4–12.5	7.8–12.8	
Pregnancy and birth						
Gestational age						
Median/Q1–Q3, weeks	40/39–41	40/39–40	0.58			
Birth weight, *n*/% of total						
<2500 g	19/4.0	30/6.0				
2500–4499 g	441/93.8	460/92.0	0.38			
>4500 g	10/2.1	10/2.0				
Birth length						
Median/Q1–Q3, cm	52/51–53	52/50–53	0.37			
Mother’s age at child’s birth						
Median/Q1–Q3, years	28/25–31	28/25–31	0.84			
Season and time period of blood sampling						
Season, *n*/% of total ^4^						
Winter	104/22.1	106/21.2		117/25.4	118/26.0	
Spring	112/23.8	125/25.0	0.49	115/25.0	116/25.6	0.11
Summer	113/24.0	137/27.4		105/22.8	126/27.8	
Autumn	141/30.0	132/26.4		123/26.7	93/20.5	
Time period, *n*/% of total ^5^						
1. 1981–1987 or 1997–1999	177/37.7	171/34.2		143/31.1	210/46.4	
2. 1987–1991 or 2000–2002	152/32.3	177/35.4	0.48	94/20.4	45/10.0	<0.0001
3. 1991–1999 or 2003–2005	141/30.0	152/30.4		223/48.5	198/43.7	

^1^ When comparing groups, the statistical tests used are chi-square test for categorical, and Mann-Whitney *U* for numerical variables; ^2^ For the newly diagnosed cohort this equals the age at blood sampling for both patients (within 3 months after onset of childhood T1D) and siblings; ^3^ First and third quartile; ^4^ Winter (December through February), spring (March through May), summer (June through August) and autumn (September through November); ^5^ The first time period represents the birth cohort and the second represents the newly diagnosed cohort. T1D, childhood type 1 diabetes.

**Table 2:** Absolute levels of peripheral immune mediators stratified by cohort and case status.

Peripheral Immune Mediators	Birth Cohort		Newly Diagnosed Cohort	
	Patient	Control	Patient	Sibling
	(*n* = 470)	(*n* = 500)	(*n* = 460)	(*n* = 453)
IL-1β				
Median/Q1–Q3, ng/L	45.1/22.8–80.0	40.3/21.3–77.1	23.0/10.3–71.3	18.2/8.1–58.9
IL-4				
Median/Q1–Q3, ng/L	20.1/12.4–31.0	20.7/12.8–31.7	9.5/2.0–21.7	8.4/2.0–17.2
IL-6				
Median/Q1–Q3, ng/L	33.7/21.3–60.0	37.5/22.1–65.5		
CXCL8				
Median/Q1–Q3, ng/L	85.2/58.7–138.4	89.8/63.7–138.3	2517.3/433.5–4914.1	1923.5/305.6–4593.0
IL-10				
Median/Q1–Q3, ng/L	242.9/64.5–793.8	245.3/72.5–721.2	47.2/25.8–92.8	44.2/20.9–79.7
IL-12				
Median/Q1–Q3, ng/L	94.8/56.5–147.8	92.9/52.5–146.5	27.5/12.3–56.1	21.7/10.2–45.6
IL-18				
Median/Q1–Q3, ng/L			183.8/119.8–285.9	142.4/104.0–212.0
IFNγ				
Median/Q1–Q3, ng/L	34.7/17.1–61.9	39.8/19.8–65.2	106.1/57.9–211.3	93.9/50.1–177.3
TNFα				
Median/Q1–Q3, ng/L	43.7/25.8–67.7	42.7/26.1–68.0	69.4/26.3–134.8	65.0/16.1–131.9
TGFβ				
Median/Q1–Q3, ng/L	927.2/603.5–1385.3	1006.6/687.7–1414.9	233.6/142.6–341.3	166.9/104.2–256.4
Leptin				
Median/Q1–Q3, ng/L	3079/1953–4678	3143/2022–4588	368.1/174.5–772.1	430.6/192.1–930.7
Adiponectin				
Median/Q1–Q3, mg/L	13.4/9.3–20.1	13.3/9.1–19.8	14.9/11.0–19.5	14.4/11.1–19.7
CRP				
Median/Q1–Q3, mg/L	0.9/0.3–2.0	1.0/0.4–2.0	0.04/0.01–0.16	0.02/0.01–0.09
MBL				
Median/Q1–Q3, mg/L	0.6/0.2–1.0	0.5/0.18–1.1	1.9/0.7–3.3	1.2/0.5–2.3
sTREM-1				
Median/Q1–Q3, ng/L	2868.5/1533.4–4930.9	2693.5/1524.9–5180.5	3368/244–16411	1085/244–9442
CCL2				
Median/Q1–Q3, ng/L			303.3/174.3–725.6	261.1/146.1–683.5
CCL3				
Median/Q1–Q3, ng/L			234.5/120.4–520.3	202.2/107.7–517.7
CCL4				
Median/Q1–Q3, ng/L			330.7/187.1–704.5	320.1/186.8–739.6
CCL5				
Median/Q1–Q3, ng/mL			10.8/6.7–29.2	13.7/7.2–36.3

IL, interleukin; IFNγ, interferon gamma; TNFα, tumor necrosis factor alpha; TGFβ, transforming growth factor beta; CRP, c-reactive protein; MBL, mannose-binding lectin; sTREM-1, soluble triggering receptor expressed on myeloid cells-1, CXCL, chemokine (c-x-c motif) ligand; CCL, chemokine (c-c motif) ligand.
